# Development of general expertise factor and analysis of its relation to decision-making behavior of gymnastics coaches

**DOI:** 10.3389/fpsyg.2026.1892922

**Published:** 2026-08-11

**Authors:** Edvard Kolar, Saša Veličković, Rado Pišot, Maks Tušak

**Affiliations:** 1 Science and Research Center Koper, Institute for Behavioral Economics, Koper, Slovenia; 2 Faculty of Sport and Physical Education, University of Niš, Niš, Serbia; 3 Science and Research Center Koper, Institute for Kinesiology Research, Koper, Slovenia; 4 Surgical Clinic, University Medical Center Ljubljana, Ljubljana, Slovenia

**Keywords:** decision-making behavior, demographic characteristics, expertise, gymnastics coaches, professional characteristics

## Abstract

**Introduction:**

Coaches may be regarded as highly qualified professionals in elite sport, possessing extended experiences, specialized tertiary education and domain-specific credentials, which designate them as experts in their particular sport discipline. They are a central figure in the development of an athlete's results. In the present study, we developed the concept of the General Expertise Factor (GEF) and investigated the associations between the level of expertise of gymnastics coaches and certain demographic and professional characteristics, as well as their decision-making (hereinafter DM) behavior.

**Methods:**

To reveal the structure of the DM style, a general DM style questionnaire was used, to which questions on the demographic and professional characteristics were added. Pearson's correlation coefficient was used to identify the correlations, and multiple regression analysis was employed to examine causal associations between measured parameters. In addition, ANOVA was used to detect differences in all measured parameters between groups of coaches defined according to different expertise criterions.

**Results:**

The results indicated that development of GEF is based on the premise that expertise in a particular field is multidimensional, whereby dimensions or criterions of domain-specific explicit knowledge, experience, and confirmed results must be simultaneously achieved for an individual coach to be recognized as an expert of the highest rank. Statistically significant differences between individual expert groups of coaches were found for all three selected expertise criteria. Moreover, the comparation of the resulting quality groups of coaches according to their average DM behavior reviled that the groups differ significantly in the use of rational DM style, with rationality in DM consistently increasing in line with the increasing competence or expertise of the representatives of each quality group.

**Conclusions:**

Based on the findings we can conclude that the developed GEF well stratifies gymnastics coaches into different levels of competence and expertise and that rationality is one of the important factors that, together with knowledge, experience and achieved success, represents a defining element of coaching expertise. The developed GEF model was also checked for compliance with other, already established theories and models in the fields of expertise and DM, where a high level of consistency was found.

## 1 Introduction

Research consistently shows that athletic performance depends on the complex interaction and interplay between (1) genes and (2) opportunities to express those gens through exposure to right environment and practice opportunities (e.g., Bloom, 1985; Johnson et al., 2010; Williams et al., 2018). Based on expert reviews in 1st edition of *Cambridge Handbook of Expertise and Expert Performance* edited by Ericsson et al. (2006), Dobbs (2006) argues that contemporary understanding of expert performance suggests, that it depends on or is enabled by: (1) 1% inspiration (innate qualities), (2) 29% good teaching and encouragement (environment), and (3) 70% perspiration (deliberate practice). The purpose of this article is not to discuss the importance of nature or nurture in achieving an athlete's final result, but rather to identify and understand which characteristics influences and shapes the expertise of coaches and how is coaches' decision-making (hereinafter DM) behavior related to them in the development of athletes' high-level performance in general and in gymnastics in particular. Accordingly, the role of coaches in the process of developing athlete performance can be found in the two factors of the aforementioned Dobbs (2006) equation. First, coaches represent an enablers of deliberate practice process, and second, they represent the most important part of the athlete's environment, as in the direct pedagogical (training) process they facilitate the development of all aspects of the athlete's preparation, which in the narrow sense enables athletes to participate and perform in a particular sport.

Deliberate practice was defined as specific, purposeful, and challenging practice of an aspect of performance that requires (1) improvement, (2) explicit goals, and (3) effortful repetition of activity (4) supported by feedback (Williams et al., 2018). Since the seminal work of Ericsson et al. (1993), several studies have been conducted to prove or disprove the theory of deliberate practice in various fields of human activities. Many of applied studies have also investigated the effects of deliberate practice in the field of sports training, with positive effects being demonstrated both (1) in terms of the greater number of practice hours spent achieving elite vs. sub-elite levels of performance (e.g., Hodges and Starkes, 1996; Helsen et al., 1998; Law et al., 2007; Berry et al., 2008; Moesch et al., 2011; Roca et al., 2012; Baker and Young, 2014), as well as (2) the positive effects of more organized training and individual instructions from coaches on athletes' performance in different sports (e.g., Baker et al., 2003; Weissensteiner et al., 2008; Coughlan, 2016), including those classified as conventional sports (Starkes et al., 1996; Deakin and Cobley, 2003; Law et al., 2007). A growing body of research also shows that performance improvement as a function of continued training is often not monotonic and shows plateaus in individuals' performance (Gray, 2017) that are not immutable and can be overcome with changes and coach-led training (deliberate practice). Likewise, Ericsson and Charness (1994) clearly stated that

“*independent learning has most of the characteristics of deliberate practice but is probably not as effective as individual learning guided by a qualified teacher*.”

Based on decades of research in the field of expertise development, Ericsson and Harwell (2019) emphasized that deliberate practice differs qualitatively from most other forms of practice and point out four criteria's that outline the role of coaches in the sports training process: (1) that practice involves individual coaching of the athlete by a well-trained (expert) coach who can assess which aspects of the athlete could improve and can formulate practical instructions for effective improvement; (2) that the coach must be able to communicate the goal that the athlete must achieve; (3) that the coach can describe a practice activity to achieve the defined performance goal and that this activity allows the athlete immediate feedback on a given attempt; and (4) that the coach provides additional feedback on how well the athletes have achieved the goals, which can help them improve their mental representations so that they can reliably notice differences that have not yet been successfully achieved. Once current goals are achieved, (5) the coach identifies new goals and associated training that allow the athlete to continue to improve their performance through additional deliberate practice. To achieve all this and meet all the aforementioned requirements, coaches must develop extensive sport training-related theoretical knowledge and gain a lot of domain-specific practical experiences. So, they need to develop expertise and become experts in the field of sports training.

Sports training in gymnastics is a complex process in which the central place is occupied by the technical preparation of the athlete, which is connected with the (1) process of recognizing the technique of gymnastic movements (elements) and the (2) methodology of learning the elements. Technical preparation (elements learning) is therefore a key element of planning, implementation, control and evaluation of the entire gymnast's preparation and also the most important component of the expertise of a gymnastics coach (Kolar et al., 2006). Williams et al. (2018) distinguish between learning and performance, emphasizing that learning cannot be directly observed, but can be inferred from changes in behavior that can be observed by measuring performance in practice and competition. They also state (2018), that it has been shown that performance in practice is better when coaches provide (1) instructions explicitly and frequently (either visual or verbally), (2) use practice schedules that encourage repetitive, blocked practice of a single skill, and (3) provide copious amounts of feedback. In this sense, we can see that the requirements of technical preparation represent the basis on which coaches guide the processes of physical, psychological and all other aspects of the preparation of an athlete in gymnastics (Kolar et al., 2026). Furthermore, Smith (2003) notes that,

“*the comprehensive monitoring* (and evaluation) *of athletes is necessary in order to allow a coach to make informed* (rational) *decisions regarding the effects and consequent planning of training*.”

Based on this, we can conclude that the process of learning the elements guides the processes of strategic, operational and tactical DM of coaches in gymnastics (Kolar et al., 2025a).

Accordingly to the above, the results of athletes in elite sports depend largely on the expertise and decisions their coaches make during the training and competition process. Different authors argue that coaching is, fundamentally a DM process (Abraham and Collins, 2011; Lyle and Muir, 2020), while coaches' DM represent defining element of coaching expertise (Kaya, 2014; Harvey et al., 2015; Coutts, 2017; Till et al., 2019; Post and van Gelder, 2023). Some coaches' decisions are result of deep thinking based on the effort invested in finding information, analysis and long discussions and others are again quick and sudden and prompted by various situations that occur in training and competition settings and require quick reactions. From this perspective, different type of decision has its own unique role, meaning and purpose in the process of competitions and sports training, but all of them are accumulated and evaluated by the athlete's final performance, which is measured by the result in the highest-ranking competitions. Regardless of what decision a coach must make at a particular stage of an athlete's career development, the quality of that decision will largely depend on the coach's level of knowledge and experience, i.e., the level of expertise achieved.

In this study, we developed the concept of the General Expertise Factor (GEF) and investigated the associations between the level of expertise of gymnastics coaches and certain demographic and professional characteristics, as well as their DM behavior measured with the General Decision-Making Style Inventory (hereinafter GDMS).

## 2 Literature review

Coaches may be regarded as highly qualified professionals in elite sport, possessing specialized tertiary education and domain-specific credentials, which designate them as experts in their particular sport discipline (Lyle and Muir, 2020). Namely, Quinaud et al. (2026) find out that maturity, practical experience, and continuing education proved to be essential for the development of expertise in sports coaching. Ericsson (2018a) wrote that an expert is someone who is highly qualified and well-versed in a particular field, or someone who is widely recognized as a reliable source of knowledge, techniques, or skills, whose judgments (and decisions) are recognized by the public or by peers as relevant and correct. However, Ericsson (2018b) himself notes that extensive experience in a certain field does not always lead to an expert level of achievement, as it is not always associated with deliberate improvement in performance. Accordingly Kolar et al. (2025a) supplemented the definition of expertise with an additional criterion related to the “*the constant achievement of top results at the highest level of international competitions by the athletes they coach*,” which is also consistent with Collins and Evans (2018) so-called “*periodic table of expertise*” where authors identify meta-criteria's for identifying experts including (1) credentials (explicit formal knowledge), (2) experience (implicit knowledge), and (3) track-record (achieved results). In a same way also Starkes' (1993) defined that the consistent superior athletic performance over an extended period of time is a fundamental criterion of expert performance in sport.

Côté et al. (1995) researched an expert high-performance gymnastics coaches and wrote that when expert coaches estimate a gymnast's potential, they construct a mental model of what needed to be done to develop that particular gymnast. This model is then used as a basis to define which (technical) knowledge was important for use in the competition, organization, and training process components. This mental model is defined as the coach's estimation of a gymnast's potential and then served to establish the actions for developing that particular gymnast. They argue that large arsenal of coaches' knowledge, organized hierarchically through the different properties, categories, and components which allows expert coaches to rapidly assess situations that do not fit their mental model and, consequently, make the appropriate changes (Côté et al. (1995)). The authors used several selection criteria to identify expert high-performance coaches, including: (1) at least 10 years of coaching experience, (2) having coached at least one international-level gymnast and two national-level gymnasts in the senior category who earned a spot on a team at an international competition or national championship by the national federation, and (3) are being recognized by the national federation as one of the best national coaches in training top-level gymnasts (Côté et al. (1995)).

Coaches are also often understood as managers of the training process, overseeing planning, organization, implementation, and evaluation (Nash and Collins, 2006; Naylor, 2006). They coordinate experts and athletes and delegating tasks to optimize training process (Kolar et al., 2025a). The aforementioned sets of the coach's managerial tasks in a sports training process represent the substantive part of the coach's work, while the basic method of his work is making decisions (Abraham et al., 2006; Wilson and Kiely, 2023; Kolar et al., 2025a). Accordingly, the process of creating a mental model of athletes' development defined by Côté et al. (1995) requires the collection of data, assessments, and predictions, which form the rational-analytical basis for making decisions that coaches later implement in competition, organization, and training processes.

DM is according to Daft (2010), the process of identifying problems and opportunities and finding a solution, the result of which is a decision. The decisions we make are always burdened with consequences that are an inevitable result of our DM process. Just as Kahneman et al. (2021) distinguish between judgment and the decision that should follow the judgment, Klein and Mathlie (1992) also emphasize that it is necessary to distinguish between the concepts of the DM process and the result, which is reflected in the decision made. The DM is a mental (psychical) process and is defined as a more or less systematic process of collecting and organizing knowledge, in which (1) we should obtain enough information to make an appropriate decision, (2) reduce the possibility of overlooking something essential, and (3) be aware of the risks and (4) consequences of the decision. The DM process undoubtedly has a major impact on the quality of decisions, and consequently on success, because if we follow a regulated process in DM, the probability that the decision will be appropriate is greater than if the DM process is less regulated or even random (Rozman and Kovač, 2012). Skinner (1999) writes that a decision is a conscious and irreversible allocation of resources with the aim of achieving desired goals.

DM largely depends on the cognitive abilities that an individual uses when evaluating various DM-relevant content for which is responsible his cognitive style. Cognitive style has historically been associated with consistency in an individual's way of cognitive functioning, especially with regard to (1) acquiring and (2) processing information (Ausburn and Ausburn, 1978). Regardless of the fact that, based on contemporary research, it is possible to identify several types of DM and the implementation of judgment processes at the collective level (Davenport, 2013), DM processes at the individual level can be classified into two fundamental forms or cognitive styles, i.e., intuitive and analytical. Therefore, according to Kahneman (2017)'s definition, the two cognitive styles are labeled as System 1 (intuitive-experiential cognitive style), which is defined by immediate automatic decisions based on the feelings of decision-makers, which are the result of intuitive, experiential or impulsive reasoning, and on the other hand, DM processes, called System 2 (rational-analytical cognitive style), in which decisions are made based on the application of the results of theoretical knowledge and in-depth analyses of DM issues and are thus the result of logical, rational and reflective reasoning.

The cognitive style of an individual can therefore be understood as a latent, hierarchically superior cognitive dimension (Allinson and Hayes, 1996; Hodgkinson and Sadler-Smith, 2003; Cools et al., 2006; Epstein, 2015) that develops in two dominant styles of cognitive functioning. The aforementioned cognitive styles (analytical and intuitive) can be given different names depending on the definitions of different authors, but they are said to play a decisive role in the manifestation of DM styles, which are manifested as specific behaviors of individuals in DM processes. DM styles are outwardly manifested as an analytical or intuitive approach to solving DM problems or in the form of various combinations of the two aforementioned “pure” cognitive styles. DM styles of individuals represent a manifest level of judgment and DM and thus shape the DM behavior of each individual (Kolar, 2026).

Many authors have dealt with the definition of DM styles. It is characteristic of research in the field of DM styles that, in addition to defining what DM styles are, authors often also create different models and constructs that include a different number of DM styles (Harren, 1979; Rowe and Mason, 1987; Scott and Bruce, 1995; Nygren, 2000). The most commonly used questionnaire for assessing DM styles (Berisha et al., 2018; Urieta et al., 2021) is the questionnaire developed by Scott and Bruce (1995), who defined DM styles as “*a learned response or behavioral pattern of an individual faced with a DM situation*.” They argue that it is not a personality trait, but a tendency to react in a specific way in a DM situation, with the characteristics of the situation itself also having a great influence. The authors also state that individual DM styles are not mutually exclusive and that individuals do not rely exclusively on one DM style (Scott and Bruce, 1995). The results of their study showed that the structure of DM styles is formed by five styles and that the questionnaire thus designed can be used regardless of the context or DM situation. The five DM styles included in their GDSM are defined as: (1) rational, (2) intuitive, (3) dependent, (4) spontaneous, and (5) avoidant.

Over the past 40 years, the DM of sports coaches has been the focus of interest of a large number of authors from different professional, geographical and cultural backgrounds. Research on coaching DM deals with this phenomenon from various perspectives and in different contexts, often in connection with other psychosocial aspects, demographic and professional characteristics of coaches, and in relation to the impact of coaching DM on athlete satisfaction, motivation, wellbeing, and performance. Research in this area can therefore be framed in several content areas, such as (1) leadership styles of sports coaches in the context of DM behavior (e.g., Jin et al., 2022; Jawoosh et al., 2022), (2) Klein's (2015) natural DM paradigm (NDM) and the recognition-based DM model (RPD) in sports coaches' DM processes (e.g., Abraham and Collins, 2015; Lyle and Muir, 2020), (3) the concept of professional judgment and DM (PJDM) of sports coaches' DM (e.g., Collins et al., 2016), (4) DM styles as a concept of sports coaches' DM behavior (e.g., Giske et al., 2013; Noh et al., 2018; Kolar et al., 2025a), and (5) other aspects and classifications of coach DM (e.g., Post and van Gelder, 2023), where the authors provide a typology of different types of decisions made by coaches (for a better overview of the concepts, see Kolar et al., 2025a). In accordance with the concept and objectives of the existing study, only the results of research in which the authors used the GDMS questionnaire (Scott and Bruce, 1995) for a theoretical or empirical explanation of coaches' DM behavior in relations to several aspects of sport training will be presented in more detail below.

Kolar et al. (2025a) in their study developed a conceptual framework of coaches' DM in conventional sport (e.g., gymnastics, figure skating, diving) which covers the most comprehensive range of situations that can arise in the competition and/or training process and possible ways of dealing with them, which should result in different types of decisions and characteristic of coaches' DM behavior. The developed conceptual framework foresees three types of decisions (strategic, tactical and operational), which should have a different role in the comprehensive process of sports training. In order to define the types of decisions, the (1) expected time frame of the validity, (2) time impact and (3) level of urgency of decisions made was mainly used. Authors point out that foresees three types of decisions should have a (1) different role in the comprehensive process of sports training, should be (2) carried out on the basis of different cognitive processes, (3) manifested in the forms of different DM style structure and (4) should be enforced by using different leadership styles. Therefore, as authors wrote, if we are aware of the level of expert knowledge and the amount of domain-specific experiences of the coaches and we find out (measure) their DM styles structure, the conceptual framework gives us relatively precise instructions as to which coach entrust the management of athletes in the processes of training and competitions (Kolar et al., 2025a).

Giske et al. (2013) investigated the DM styles of football coaches in relation to their coaching experience at elite and non-elite levels of coaching and their level of playing history. The results of their study show that football coaches mostly use a rational or intuitive DM style and almost no avoidant DM style, and that coaches with higher expertise in a specific field of coaching statistically significantly use a more rational and intuitive DM style than non-experts. In addition, coaches with experience at elite level show a statistically significantly higher value of using intuitive and rational DM styles than coaches who do not have this experience or have it to a lesser extent.

Another study conducted by Noh et al. (2018) investigated the relationship between the DM styles of football club coaches with basic psychological needs and intention to continue training. The results of this study showed that rational and intuitive DM styles of coaches have a positive effect on the basic psychological needs of participants, while dependent and avoidant DM styles of coaches have a negative effect on their basic psychological needs. In addition, this study also showed that rational and intuitive DM styles of coaches have a positive effect on the intention to continue training of participants, while avoidant DM styles of coaches have a negative effect on their intention to continue training.

A study by Kolar et al. (2025b) investigated the behavior of Serbian gymnastics coaches and determined the average structure of DM styles, how DM styles are related to certain demographic and professional characteristics, and what are the differences in DM styles between more and less experienced Serbian coaches. The results showed that coaches use a combination of all five DM styles, mostly rational, dependent, and intuitive DM styles. Based on the determined average structure of DM styles, the authors conclude that Serbian gymnastics coaches are mostly rational decision-makers who increase their rationality of DM by seeking advice, opinions, and knowledge from colleagues, and that more experienced coaches can make decisions more independently and also faster when situations are urgent or time-limited.

In all discussed studies, the structure of DM styles within all formed samples (whole, experts, non-experts...) was very similar. Coaches show the highest proportion of the use of functional DM styles (1) rational and (2) intuitive, followed by the so-called non-functional DM styles, (3) dependent, (4) spontaneous and (5) avoidant DM style. The use of functional DM styles in DM processes generally leads to correct and effective decisions, while the increased presence of non-functional styles in the overall structure of coaches' DM styles could indicate the risk that their DM behavior often led to negative results and wrong decisions (Mitchell et al., 2010; Faletič and Avsec, 2013). In the case of established connections between individual criteria of expertise, Kolar et al. (2025a) in their developed conceptual framework of DM of coaches in conventional sports predicted, that explicit expert knowledge is decisive for judgment in DM process when making strategic and tactical decisions, while domain-specific experience is especially important in judgment in making operational decisions. Furthermore, Kolar et al. (2025b) reveal that coaching experience is statistically significantly positively associated with dependent and spontaneous DM styles and Giske et al. (2013) reveal that football coaching experience is statistically significantly positively associated with rational and intuitive DM styles.

## 3 Research design and method

### 3.1 Participants

The sample consisted of 196 gymnastics coaches with competitions experience (age: 36.08 ± 12.57 years) from Serbia (44; 22.4%) and Slovenia (152; 77.6%). Measurements were conducted during national coaching seminars in Slovenia (January 2025) and Serbia (August 2023). The upper age limit was 79 years, whereas the lower age limit was 18 years. The most experienced coach had 50 years and the least experienced 1 year of work experience (13.481 ± 0.41 years). The sample consisted of 50 men (25.5%) and 146 women (74.5%). The level of education of the coaches included in the sample is distributed across the entire spectrum from incomplete secondary school to a PhD level, with the average value being slightly higher than the 1st Bologna level (bachelor's degree). All subjects participated in the study anonymously, voluntarily and without any compensation.

### 3.2 Instrument

The DM style was measured with the use of the GDMS (Scott and Bruce, 1995), which was translated into the Slovenian (Avsec, 2012; Faletič and Avsec, 2013) and Serbian language (Kolar et al., 2025a). The GDMS questionnaire measures five different DM styles: rational, intuitive, dependent, avoidant and spontaneous. The questionnaire consists of 25 items (5 for each DM style) ranging on a five-point Likert scale from strongly disagree (1) to strongly agree (5). A total score for each of the five DM styles was obtained by summing the item scores that measured each DM style, and the score ranged from 5 to 25. GDMS scales have previously shown good psychometric characteristics on different samples (managers, students, the general population, military officers, sport managers, sport coaches and others) from different countries (Scott and Bruce, 1995; Thunholm, 2004; Spicer and Sadler-Smith, 2005; Curşeu and Schruijer, 2012; Avsec, 2012; Giske et al., 2013; Noh et al., 2018; Alacreu-Crespo et al., 2019; Kolar and Tušak, 2022; Kolar et al., 2025a). In this study, the alpha coefficients of the scales ranged between 0.500 (spontaneous) and 0.849 (avoidant; Table 1). The Cronbach's alpha for the whole GDMS is 0.715, which is a good indicator of internal consistency. The reliability coefficient of the questionnaire items for the spontaneous style can be assessed as sufficient, while the other coefficients indicate moderate to robust internal consistency (Taber, 2018). However, results involving the spontaneous subscale should be interpreted with caution, as potential measurement error may have weakened its correlations and reduced the likelihood of detecting associations with expertise variables. The general information questions about demographics characteristics (gender and age) and professional (expertise) characteristics (experiences, level of education, category of athletes that they coach, status in national coaching hierarchy and success) of a coach were also added.

**Table 1:** Descriptive statistics and internal consistency of DM styles.

DM styles	*N*	Min	Max	Mean		Std. deviation	Skewness	Kurtosis	Cronbach α
				Value	% of max				
DMSR	196	12	25	20.20	80.82%	2.556	−0.347	0.123	0.753
DMSI	196	9	25	17.62	70.49%	2.900	0.107	0.101	0.669
DMSD	196	7	25	17.42	69.67%	3.374	−0.263	0.286	0.762
DMSA	196	5	24	11.25	46.88%	3.961	0.512	−0.045	0.849
DMSS	196	4	22	13.52	61.46%	2.655	−0.057	0.773	0.500

DMSR, rational style; DMSI, intuitive style; DMSD, dependent style; DMSA, avoidant style; DMSS, spontaneous style.

### 3.3 Statistical analyses

Statistical data processing was carried out using the Statistical Package for the Social Sciences 29 (IBM SPSS Inc., Armonk, NY, USA). The internal consistency of the overall scale and subscales of GDMS was measured using Cronbach's alpha coefficient. Pearson's correlation coefficient was used to identify the association between DM styles, demographic and professional characteristics of the sample as well as to determine the relationship between expertise parameters and the developed General Expertise Factor (GEF) and DM styles with GEF. Multiple regression analysis (method enter and stepwise) was employed to examine causal associations between individual predictors of coaches' expertise and GEF and also between their DM styles and GEF. In addition, one-way analysis of variance (ANOVA) was used to detect differences in all measured parameters between groups of more and less experienced, educated, and successful coaches, and to determine differences in the use of DM styles among the four groups of coaches developed based on the GEF equation.

## 4 Results

Table 1 shows descriptive statistics for five DM styles. The average values of the individual DM style use (Mean/Value) were calculated from the scores dedicated to items assigned to an individual DM style (Scott and Bruce, 1995). Average share of the individual DM style use (Mean/% of maximum) in relation to the maximum possible total value of the sum of items of the individual DM style (maximum = 25) was also calculated for each DM style.

The structure of the DM styles (Table 1) revealed that our sample of gymnastics coaches on average most often use the rational and almost at the same level intuitive and dependent DM styles. They are followed by spontaneous DM style, while coaches use avoidant DM style the least often. The same or similar results on the structure of DM styles have also been found in different samples of sports coaches in other studies (Giske et al., 2013; Noh et al., 2018; Kolar et al., 2025a). The average structure of DM styles also reveals that studied sample of coaches achieve a significantly high proportion of use of the rational DM (80.8%), as well as high proportions of use of intuitive (70.5%) and dependent (69.7%) DM styles, while the spontaneous style is used occasionally (61.5%). Avoidant style is rarely used in the training process (46.9%), but the values in our sample are slightly higher than in the samples studied by Giske et al. (2013) (DMSA = 10.90) and Noh et al. (2018) (DMSA = 10.95).

The analysis of the relationship between the measured demographic and professional variables showed that the performance (success) of coaches (Table 2), measured by the performance of their athletes, in terms of participation and/or medals won in national (national championship) and international competitions (World Cup, European Games, European Championships, World Championships and Olympic Games), is statistically significant correlated with all measured demographic and professional parameters. The associations show that coaches who achieve higher levels of performance are male, older, more experienced and educated, coach higher categories of athletes (e.g., seniors) and are at a higher level (status) in the club/national coaching hierarchy (e.g., national team coaches). Correlation analysis also revealed that two other variables measuring the professional characteristics of the sample of coaches (experience and status) have the same number and nature of associations with the demographic and professional variables as the variable “success” and that they have a high positive correlation with the performance variable (success). All three are also statistically significantly associated with the variable measuring the level of education (education). Results support findings of other authors that coaches' functional competencies are influenced by older age, higher academic background, experiences and competitive level (Mesquita et al., 2011; Egerland et al., 2014; Mitzel et al., 2020; Quinaud et al., 2026). From the obtained connections between variables measuring professional characteristics, we can also assume that coaches who (1) are more experienced, (2) achieve better results and (3) have higher education also have a higher status in the national coaching hierarchy (status).

**Table 2:** Correlations between the DM styles, demographic and professional characteristics.

Parameter	Gender	Age	Experience	Education	Category	Status	Success	DMSR	DMSI	DMSD	DMSA
Gender											
Age	−0.172^*^										
Experience	**−0.155** ^*^	**0.793** ^**^									
Education	−0.068	**0.293** ^**^	**0.240** ^**^								
Category	−0.081	0.119	**0.256** ^**^	0.046							
Status	**−0.202** ^**^	**0.439** ^**^	**0.468** ^**^	**0.204** ^**^	**0.398** ^**^						
Success	**−0.197** ^**^	**0.245** ^**^	**0.411** ^**^	**0.154** ^*^	**0.418** ^**^	**0.495** ^**^					
DMSR	−0.068	**0.308** ^**^	**0.307** ^**^	**0.172** ^*^	0.073	**0.221** ^**^	**0.153** ^*^				
DMSI	0.098	0.075	0.095	−0.121	0.056	0.047	−0.010	**0.178** ^*^			
DMSD	0.114	−0.139	−0.087	0.063	0.035	−0.058	0.021	**0.191** ^**^	−0.012		
DMSA	0.040	−0.078	−0.054	−0.065	0.010	−0.089	−0.054	**−0.225** ^**^	0.048	**0.382** ^**^	
DMSS	0.013	0.076	0.051	**−0.156** ^*^	−0.020	0.009	−0.087	**−0.161** ^*^	**0.413** ^**^	−0.121	0.071

^*^Correlation is significant at the 0.05 level (2-tailed).

^**^Correlation is significant at the 0.01 level (2-tailed).

Statistically significant correlations are indicated in bold.

An examination of the relationship between the demographic and professional characteristics of coaches from our sample, and the DM styles they use in the training and competition process (Table 2), reveals that age, experience, education, status, and success are statistically significantly positively corelated with the rational DM style (DMSR) and that education is statistically significantly negatively corelated with the spontaneous DM style (DMSS). Other three DM styles are not statistically significantly associated with any of the variables used to measure the demographic and professional characteristics. Statistically significant positive correlations between the rational and intuitive DM style (DMSI) were expected since both styles are so-called functional styles that lead to positive outcomes (Faletič and Avsec, 2013) and repeatedly showed an associated with positive personality traits (e.g., Betsch, 2004; Narooi and Karazee, 2015; Bayram and Aydemir, 2017; Alacreu-Crespo et al., 2019; El Othman et al., 2020). Rational and intuitive DM styles can and should be used alternately in making decision in a training and competition process depending on the task or situation (Kolar et al., 2025a). The rational DM style is statistically significantly negatively related to the avoidant (DMSA) and spontaneous (DMSS) DM styles, which is also confirmed by many other studies (e.g., Scott and Bruce, 1995; Spicer and Sadler-Smith, 2005; Hariri et al., 2014), where both, in contrast to the rational DM style, are mostly also related to negative personality traits and risky behavior (e.g., Bavolár and Orosová, 2015; Bayram and Aydemir, 2017; Alacreu-Crespo et al., 2019; El Othman et al., 2020). Statistically significant positive correlation between the intuitive and spontaneous DM styles, were expected and could indicate on the same latent background of both DM styles, which could be present in the intuitive-experiential cognitive style (Alacreu-Crespo et al., 2019). Their mutual positive connection can also be understood in accordance with Thunholm's (2004) definition, which defines the spontaneous DM style as a high-speed intuitive DM style, which, according to Klein's doctrine of naturalistic DM, is used in time-limited DM situations that are under time pressure (Klein, 2015). All of the statistically significant correlations mentioned so far were in some way expected, but what is unexpected and somewhat puzzling is the simultaneous statistically significant positive correlation between the dependent and rational and dependent and avoidant (DMSA) DM styles, which will become more understandable through explanations in the discussion section.

**Figure 1:**
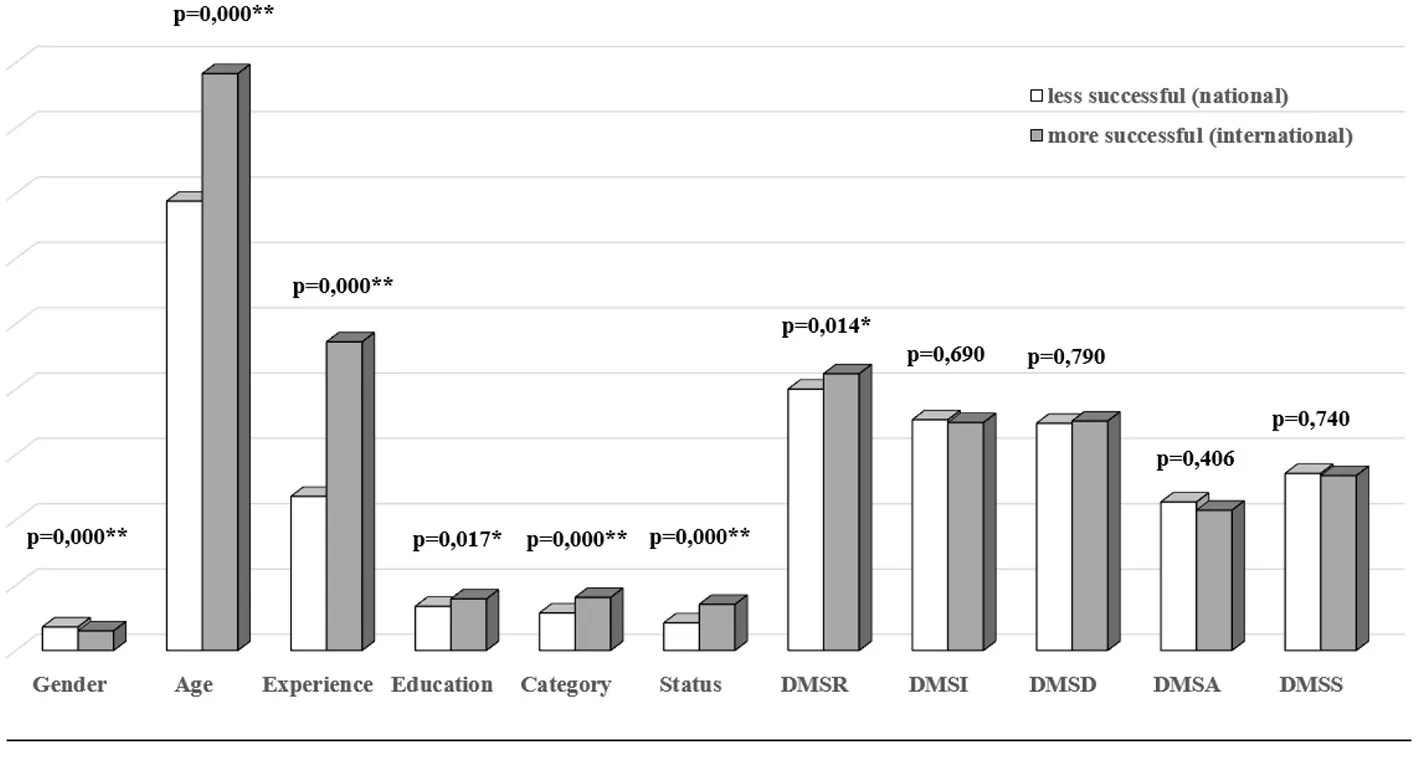
Analysis of differences between more and less successful coaches. *Correlation is significant at the 0.05 level; **Correlation is significant at the 0.01 level.

**Figure 2:**
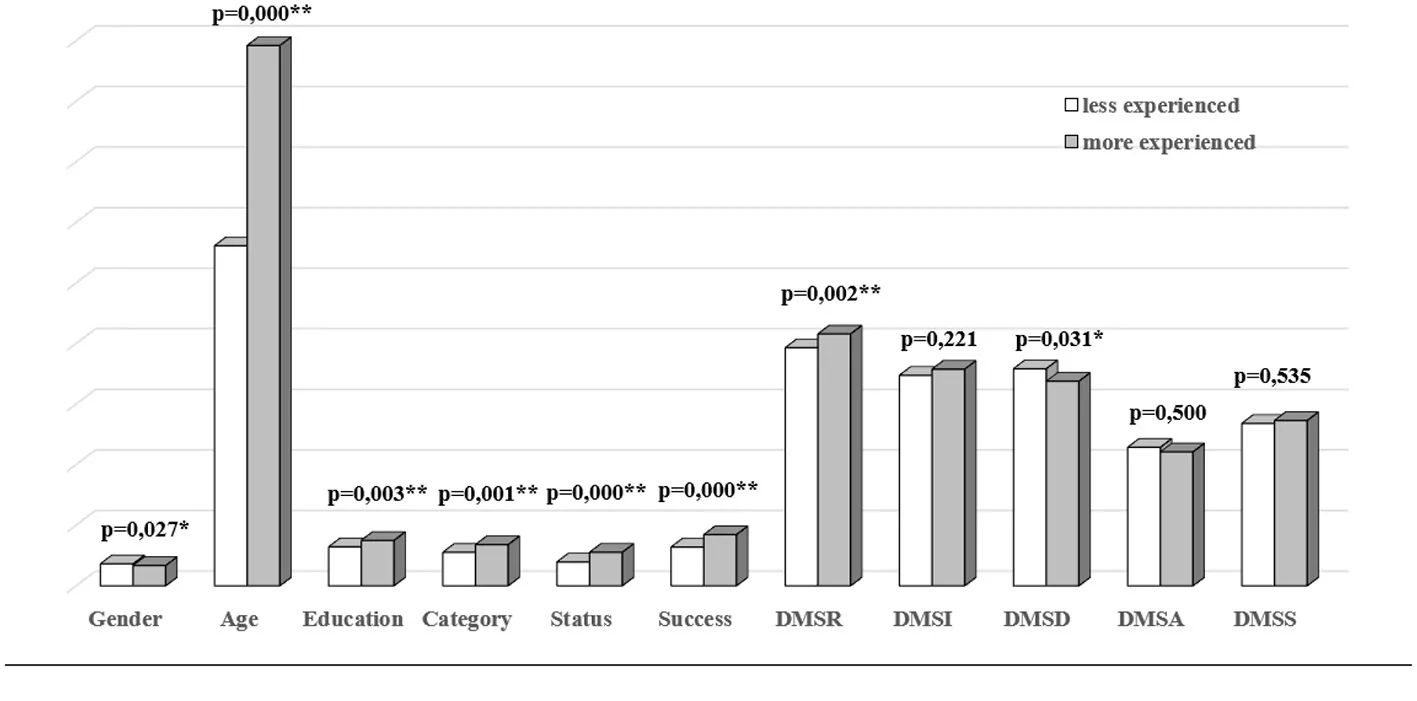
Analysis of differences between more and less experienced coaches.*Correlation is significant at the 0.05 level; **Correlation is significant at the 0.01 level.

**Figure 3:**
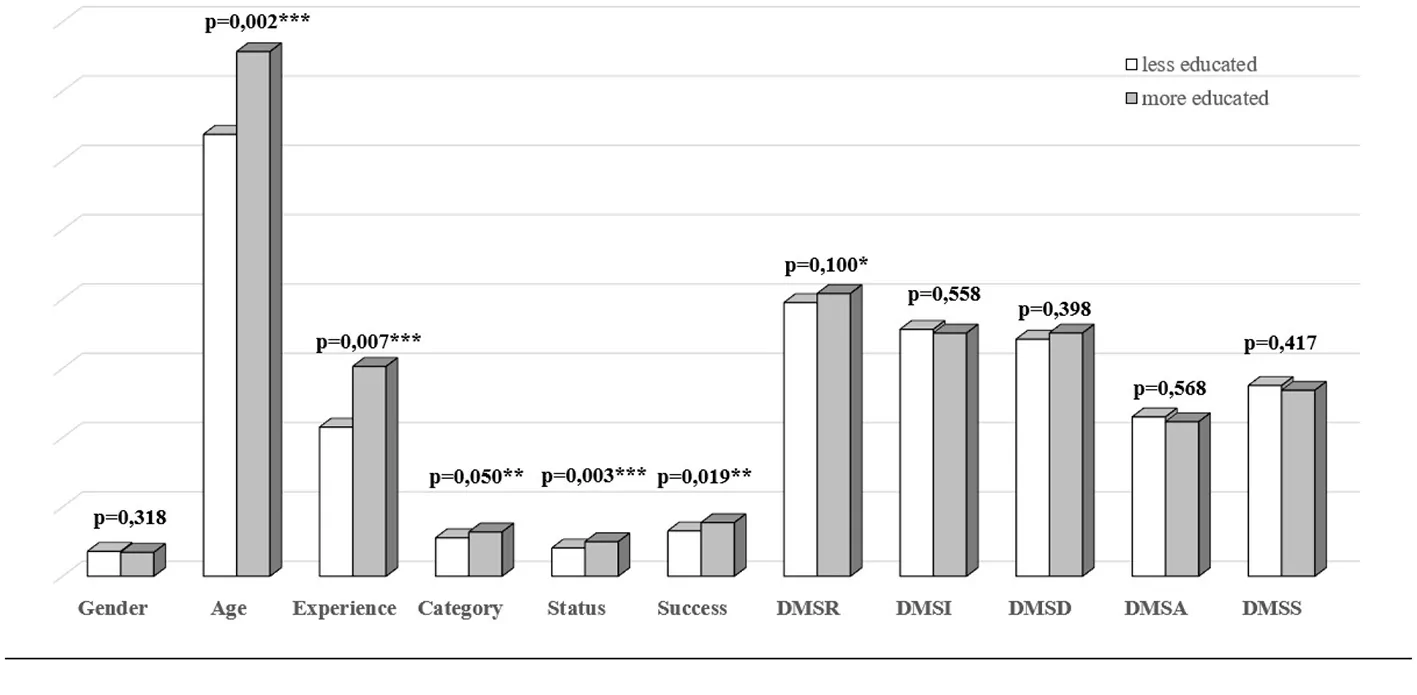
Analysis of differences between more and less educated coaches. *Correlation is significant at the 0.10 level; **Correlation is significant at the 0.05 level; ***Correlation is significant at the 0.01 level.

The analysis of the connections between the demographic and professional characteristics and the DM behavior of our sample of gymnastics coaches showed significant interrelationships between the measured parameters, where the connections of those variables that define the level of expertise in the coaching expertise (success, experience, education) proved to be particularly interesting (Starkes, 1993; Collins and Evans, 2018; Ericsson, 2018a; Kolar et al., 2025a). Consequently, in the continuation of our investigation, we divided the entire sample of coaches according to the criteria of more or less successful (Figure 1), more or less experienced (Figure 2), and more or less educated (Figure 3) coaches and investigated in which of all the measured variables (demographic, professional, DM styles) thus formed groups of coaches statistically differed from each other.

First, we divided the entire sample of coaches into two groups according to the parameter “success.” which measures the performance of coaches, by forming a group of less successful coaches (national level), whose athletes managed to qualify and/or won medal at the national championship (*n* = 162), and more successful coaches (international level), whose athletes successfully qualified and/or won a medal at the World (also Challenge) Cup, European Games, European Championship, World Championship or Olympic Games (*n* = 34) (Starkes, 1993; Côté et al., 1995; Kolar et al., 2025a). From the perspective of demographic characteristics, the results of the analysis of differences between the two groups of coaches (Figure 1) showed that the more successful coaches were statistically significantly older and that this group, on average, had a statistically significantly higher proportion of male coaches.

The groups of coaches also differ from the perspective of all professional parameters, and we can find that international coaches are statistically significantly more experienced, educated, train athletes of higher age categories and have higher positions in the national coaching hierarchy than coaches classified in the national group. From the perspective of the DM behavior of groups of coaches in relation to performance (success), we can see that they differ only in the use of a rational DM style, where more successful coaches showing statistically significant more rational DM behavior (DMSR) than coaches from the less successful group.

In the second step, we divided the sample of coaches into those with less experience, namely those with 10 or fewer years of coaching experience (*n* = 101), and those with more than 10 years of coaching experience (*n* = 95) were classified as more experienced coaches (Côté et al., 1995; Ericsson, 2018b). The results of the analysis of differences between the two groups of coaches formed in this way (Figure 2), even though that the groups are formed differently, showed similar results as the analysis of groups formed according to success parameter. The group of more experienced coaches consists of statistically significantly more male coaches and is also statistically significantly older than the group of less experienced coaches. The group of more experienced coaches is also more educated, achieves higher competition results (success), they train older age categories of athletes and has a higher status in the national coaching hierarchy. In all of the mentioned professional parameters, the differences between the groups of coaches according to experience are statistically significant. More experienced coaches also report statistically significantly more rational DM behavior (DMSR), while less experienced coaches are statistically significantly more dependent decision-makers (DMSD).

The last criterion based on which we divided the entire studied sample of gymnastics coaches into two groups was the level of education achieved. The group of less educated coaches (*n* = 58) included those coaches who had an education lower than secondary school or completed secondary school (9 years of primary plus 4 years of secondary school), while the group of more educated coaches (*n* = 138) included coaches who had completed at least the 1st Bologna level of study or higher (2nd Bologna level, scientific master or PhD) (Lyle and Muir, 2020).

Analysis of differences between the groups according to level of education showed that highly educated coaches are statistically significantly older, more experienced and more successful and, as a result, also coach statistically significantly older categories of athletes and occupy higher positions in the national coaching hierarchy. A comparison of groups of more and less educated coaches from the perspective of DM behavior reveals that the only difference between the two groups that is statistically significant at the border of 10% statistical risk level is that more educated coaches report a higher level of rational DM behavior than the less educated group of coaches.

## 5 Development of three-dimensional general expertise factor (GEF)

The correlation analysis presented in Table 2 revealed that the parameters classified by various authors (Côté et al., 1995; Collins and Evans, 2018; Ericsson, 2018a, b; Lyle and Muir, 2020; Kolar et al., 2025a; Quinaud et al., 2026) as fundamental criteria for defining coaching expertise (success, experience and education) are statistically significantly positively correlated with each other and that they are also almost entirely correlated in the same way with the other parameters that we selected to measure the professional characteristics of coaches (status, category). Correspondingly, in the continuation of the research, we divided the entire sample of gymnastics coaches according to the criteria of (1) the success or level of competitive results achieved by their athletes (Figure 1—national and international level coaches), (2) the amount of acquired coaching experience or domain-specific implicit or tacit knowledge that is intuitive and acquired through practical experience (Figure 2—more or less than 10 years of experiences), and (3) explicit knowledge or formal theoretical knowledge acquired by coaches in the process of formal education (Figure 3—more or less that completed secondary school) (Nash and Collins, 2006). Comparisons between the resulting groups of coaches showed that in all three cases the groups differed statistically significantly from each other in all four remaining measured parameters of the coaches' professional characteristics. Based on the results obtained, we can also assume that the higher value of the “category” and “status” parameters is a consequence of higher values in all three basic comparison criteria (success, experience and education), rather than vice versa. A comparison of the use of DM styles by coaches classified into groups based on the aforementioned criteria (Figures 1–3) showed that the use of the rational DM style consistently distinguishes the formed groups, with groups of more successful, experienced and educated coaches using it statistically significantly more than groups of less successful, experienced and educated coaches. Additionally, we also found that less experienced coaches use a dependent DM style more often than more experienced (Figure 2), which probably indicates that due to their lack of experience, they are less confident in making decisions and seek confirmation for them in their professional environment (Alacreu-Crespo et al., 2019; Kolar et al., 2025a).

The findings from the partial analyses based on the selected expertise criteria therefore show that, despite the fact that different groups of coaches were formed depending on the selected division criterion, the resulting groups are consistent significantly differ from each other. Partial differences between the formed groups of coaches allow us to detect differences and rank individual coaches according to each selected criterion of expertise, but they do not offer us a comprehensive assessment of overall level of expertise, which we have theoretically defined as multidimensional phenomenon. Subsequently, we combined all three criteria that define an expert coaches into an equation that, based on the previously obtained partial classifications into groups according to each criterion (Figures 1–3), allows us to calculate the value of the General Expertise Factor (hereinafter GEF). GEF is calculated based on the equation shown below in such a way, that for each individual entity of our sample (coach), the values obtained are determined separately, depending on whether they belong to the group of less successful, experienced and educated coaches (value “1” for each criterion) and/or belong to the group of more successful, experienced or educated coaches (value “2” for each criterion). The three values are thus summed into the so-called “*GEF*_*sum*_” value, the value which can range between “3” for those coaches who were classified in the group of less successful, experienced or educated coaches in all three criteria (11 + +1) and the value “6” for coaches who were in the group of more successful, experienced or educated coaches in all three criteria (22 + +2). Those coaches who were not consistently ranked in a “more” or “less” group can reach GEF_sum_ values “4” or “5,” depending on whether they were ranked once or twice in the group of more successful, experienced and/or educated coaches. The equation for calculating the three-dimensional GEF is shown in mathematical form below.


$$\begin{array}{l} GEF_{sum}=\;\begin{array}{c} Success\\ \left(value\;1\;or\;2\right) \end{array}+\;\begin{array}{c} Experience\\ \left(value\;1\;or\;2\right) \end{array}+\;\begin{array}{c} Education\\ \left(value\;1\;or\;2\right) \end{array}\\ \;=\begin{array}{c} Possible\\ GEF_{sum}\;value\\ \left(from\;3\;to\;6\right) \end{array} \end{array}$$

Three-dimensional models (success, experience and education) with two values for each dimension (less “1” and more “2”) usually form an 8-spatial scheme (2^3^) or eight possibilities for the contextual classification of evaluated entities based on three evaluation criteria's/dimensions (e.g., 11 + +1; 11 + +2; 12 + +1; 21 + +1, etc.). Given that in the presented model all three selected criterions are equally important (e.g., 12 + +1 = 11 + +2), the resulting groups are classified into four levels of expertise solely based on the sum of the values (GEF_sum_).

The resulting groups according to the level of expertise are named as “Novice” coach, “Advance” coach, “Proficient” coach and “Expert” coach. The method of forming each group and the characteristics of the expertise and competencies of the coaches classified into each group as well as the descriptive statistical characteristics of each qualitative group of gymnastics coaches from our sample are presented in Table 3.

**Table 3:** Description of groups formed based on general expertise factor (GEF) equation.

GEF groups				
No	GEF_sum_ value	Name	*N* (%)	Group description/descriptive statistics of a group
1	3	Novice	35 (17.9%)	➪ Coaches classified in the “Novice” group are those who were classified in the group “1” in all three expertise criteria (Figures 1–3) from the GEF equation, meaning that the value of their calculated GEF_sum_ is equal to 3, which represents the lowest possible value. ➪ At this level, coaches are able to understand the fundamentals of sports training in gymnastics and have the competences to manage the training and competition processes mostly under the supervision of more qualified mentors. Coaches have little experiences and knowledges and are in process to develop their coaching skills. ➪ The “Novice” group consists mostly of female coaches (29; 82.9%), with an average age of 25.7 years (± 8.05), who have an average of 4.8 years of experience (± 2.48) and have completed secondary school, who mostly coach children up to 14 years old, are on average assistant coaches in clubs and participate with their athletes in national championships or smaller international competitions.
2	4	Advanced	82 (41.8%)	➪ Coaches classified in the “Advanced” group are those who were classified in the group “2” in at least one of the three expertise criteria (Figures 1–3) from the GEF equation, meaning that the value of their calculated GEF_sum_ is equal to 4. ➪ Coaches at the “Advanced” level have gained a more thorough understanding of the theory and practice of gymnastics training and are already leading their gymnasts to higher levels of international competition. In most cases, they are able to independently plan, organize and conduct individual training sessions and have longer experience, more specific knowledge of individual areas and a deeper understanding of the role of the coach in the training and competition processes. ➪ The “Advanced” group is mainly composed of female coaches (66; 80.5%), with an average age of 32.3 years (± 10.57), who have an average of 9.55 years of experience (± 6.97) and mostly have completed 1st Bologna cycle study programs, mostly coach youth categories up to 18 years of age, are on average coaches in clubs and participate with their athletes in national championships or smaller international competitions.
3	5	Proficient	52 (26.5%)	➪ Coaches classified in the “Proficient” group are those who were classified in the group “2” in at least two of the three expertise criteria (Figures 1–3) from the GEF equation, meaning that the value of their calculated GEF_sum_ is equal to 5. ➪ Coaches at this level have a comprehensive understanding of the gymnastics training process and are able to effectively use various tools and methods of coaching practice. They also have a comprehensive understanding of the international and national competition system and confidently participate in competitions of all levels with their athletes. Mostly have extensive practical experience and well-developed domain-specific theoretical knowledge. ➪ The “Proficient” group is mainly composed of female coaches (38; 73.1%), with an average age of 43.3 years (± 11.19), who have an average of 20.12 years of experience (± 9.22) and in average completed university level study programs (4 years), mostly coach senior categories of athletes, are on average head coaches in clubs and participate and winning medals with their athletes in national championships and participate in international arena.
4	6	Expert	27 (13.8%)	➪ Coaches classified in the “Expert” group are those who were classified in the group “2” in all three expertise criteria (Figures 1–3) from the GEF equation, meaning that the value of their calculated GEFsum is equal to 6, which represents the highest possible value. ➪ The “Expert” level is the highest level of expertise. At this level, coaches have a comprehensive understanding, wide domain-specific experiences and systemic knowledge related to the preparation of athletes for the highest level of competition. They are also qualified to create innovative approaches in the development of training approaches and valid evaluation of the level of training and performance results of athletes. They are considered leaders (e.g., national team coaches) in the field of preparation and management of athletes; they plan and shape the direction of the development of sports at the national level and make the most important strategic and tactical decisions. ➪ The “Expert” group is gender balanced (14 male; 13 female), with an average age of 47.00 years (± 9.49), who have an average of 26.52 years of experience (± 8.51) and in average completed more than 4 years university level study programs (also master and PhD level), coach junior and senior national teams athletes, and winning medals with their athletes in national championships and international arena (also World cups, European Games, European and World Championships).

**Figure 4:**
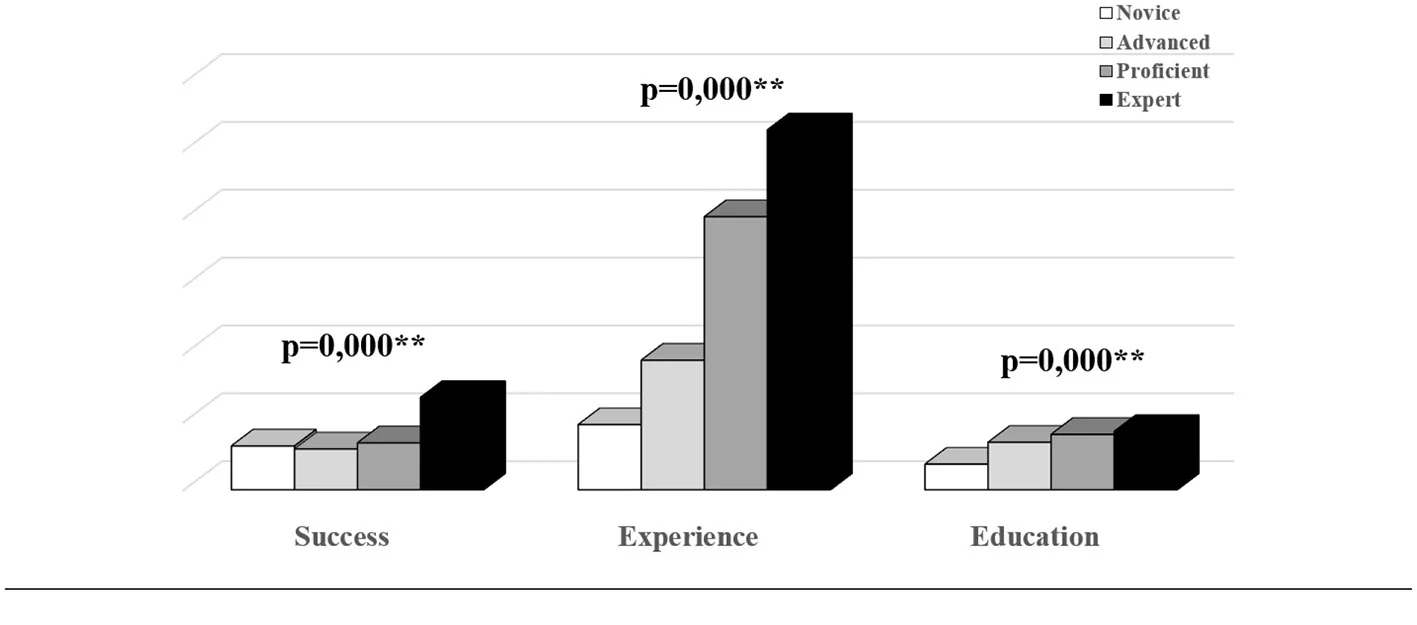
Analysis of differences in GEF criteriums between different expert level groups of coaches calculated on GEF equation. **Correlation is significant at the 0.01 level.

Resulting groups of coaches presented in Table 3 were divided in groups based on the GEF equation, represent four qualitative levels of coaches' expertise. As expected, the largest group of coaches in terms of expertise level is the “Advance” group, followed by the “Proficient” group and the “Novice” group, while the smallest in terms of number is the “Expert” group of coaches. An examination and comparison of descriptive statistics between individual groups (Figure 4) show differences between groups, indicating that the average values of the selected discrimination parameters included in the GEF equation increase or improve at each higher level. The latter indicates that the GEF factor is eligible for classification of coaches into different quality levels of expertise. Moreover, ANOVA showed that the groups of coaches formed on the basis of the GEF equation differ statistically significantly from each other (1% risk level) in all three selected expertise criteria (Figure 4), with the differences being statistically significant (1% risk level) also individually between all groups in the “experience” criterion, and with the exception between the “Proficiency” and “Expert” groups also in the “education” criterion. While for the criterion “success,” only the “Expert” coaches' group is statistically significantly different (1% risk level) from all other groups, and the “Proficiency” group is statistically significantly different (5% risk level) from the “Advanced” coaches group.

Furthermore, the correlation analysis between GEF and the criteria for classifying coaches into groups (Table 4) shows that all three criteria are highly statistically significantly associated with GEF. Correlation coefficients show also that the criterion “expertise” is most closely related to GEF, followed by the criteria “success” and “education”.

**Table 4:** Correlations between GEF and three criterions of coaches' expertise.

Parameter	Success	Experience	Education
GEF	0.584^**^	0.702^**^	0.574^**^

^**^Correlation is significant at the 0.01 level.

Following the correlation analyses, we also examined regression analyses to determine which predictor variable (experience, success, education) best explain the variance of expertise or GEF (criterion) of the selected sample of gymnastics coaches. The enter method was used as the primary theory-driven analysis to evaluate the joint contribution of the pre-defined expertise criterions. The results of the regression analysis (method “enter”) showed that all three predictors together explain 75.3% (adjusted R square) of the variance of GEF (*p* = 0.000). Stepwise regression was used only as a supplementary exploratory analysis to describe their relative incremental statistical contributions. Regression analysis using the stepwise method revealed that the largest part (49.90%) of the GFE variance is explained by the coaches' experience, the coaches' education explains 17.42% of the GFE variance, and “success” explains an additional 8.84%. Despite the statistically different contributions of individual criteria to the GEF, they were not used as weights, since in a conceptual sense, the theoretical mechanical model (GEF) treats the three dimensions as qualitatively different and at the same time necessary criteria and not as interchangeable indicators. The selection of the GEF dimensions was based on theory and was not determined by the stepwise procedure.

## 6 Discussion

According to the definition of sports training, the success of athletes depends on the efficient and effective implementation of a transformation process in which the athlete is treated as a multidimensional system that transforms through the process from a novice (when they start training in an individual sport) to an expert (when they achieve top sports results) (Kolar et al., 2025a). The success of the transformation process is measured by the achieved results (performance) at the highest level of international competitions, where the achieved result itself representing the accumulated value of the expression (realizations) of all the athlete's capabilities (knowledge, abilities, characteristics and motivation) at a certain moment (in competition), which in sports practice is referred to as “sports form”.

As presented in the introductory chapter, an athlete's performance depends largely on the (1) amount of effort the athlete puts in training process, which must be consistent with the principles of deliberate practice and (2) their immediate (primary) training environment, which is largely represented by their coaches (Bloom, 1985; Ericsson et al., 2006; Dobbs, 2006). Coaches are the central figures who manage the comprehensive process of developing an athlete's sporting performance and establish appropriate conditions for implementing deliberate practice in the process of training and competition (Ericsson and Charness, 1994; Williams et al., 2018; Ericsson and Harwell, 2019).

The dependence of the development of an athlete's results is particularly highlighted in conventional sports, where the athlete's success is encouraged primarily by the development of technical preparation (knowledge of technical skills or elements), the learning of which is a central part of the athlete's preparation from early youth until the end of their sports career. Consequently, athletes in conventional sports learn technical skills throughout their entire careers, as this allows them to increase the technical difficulty of their competitive routines, which is a fundamental pre-requisite for achieving high competitive results. Learning complex movement skills (e.g., a double straight somersault with multiple rotations around the longitudinal axis of the body), which are typically performed in the unsupported phase (flight phase), in a very limited and short time frame, at high speeds and high forces and torques acting on the body, can only be safe, efficient, and successful if supported by the coaches' (1) adequate knowledge of the technical/biomechanical characteristics of the movement, (2) appropriate methodology, (3) skilful monitoring of the execution of the movements, and (4) plenty of relevant feedback (Williams et al., 2018; Kolar et al., 2025a). All four of the above conditions are fundamental tasks of a coach in learning (pedagogical) process, when athletes are acquiring technical knowledge during process of learning new movement skills (elements). However, the success and effectiveness of coaches in performing their role largely depends on their expertise, i.e., their knowledge (explicit/formal knowledge) and their experience (implicit knowledge) (Collins and Evans, 2018; Yiannaki et al., 2026), which, if at a sufficiently high level, will eventually lead to better success of their athletes and higher levels of competitive results. Accordingly, all three of the aforementioned characteristics (knowledge, experience, and success) represent the fundamental basis for determining the expertise of trainers and defining an “expert” coach (Collins and Evans, 2018; Ericsson, 2018a; Lyle and Muir, 2020; Kolar et al., 2025a).

The developed GEF thus includes all three theoretically defined fundamental criteria for determining (and calculating) the level of expertise of coaches. The GEF equation, based on the calculated value (GEF_sum_), classifies coaches into four quality groups or four levels of expertise. Analysis of the differences between the groups thus formed shows statistically significant differences between the groups in all criteria included in the GEF equation (Figure 4), with the average values for each criterion consistently increasing from lower to higher group. Moreover, analysis of the association of GEF with the expertise criteria reveals a statistically significant association of all criteria with GEF (Table 4), and regression analysis also shows a high degree of explanation of GEF variance with the three selected criteria included in the GEF equation.

### 6.1 GEF in light of the Ericsson's theory of expertise

Although that most literature on the development of expertise and the definition of an expert suggests that 10 years of domain-specific experience are fundamental requirement to achieve the level of expertise in a certain activity (Ericsson et al., 1993; Côté et al., 1995), our research indicates a significantly more demanding and higher level of achievement of the required expertise criteria for achieving the level of “Expert” coach in gymnastics (Table 3). The multidimensional character of the developed GFE shows that a theoretically determined level (more than 10 years) of domain-specific experience (tacit/implicit knowledge) in the field of training is not sufficient to qualify an individual coach as an “Expert,” but rather expertise is also defined in terms of a higher level of education (formal/declarative/explicit knowledge) and, as can be seen from Figure 4, primarily the high level of proven competitive results (success) of the athletes they train. Namely, the analysis of individual expertise criteria reveals that the separate use of the criteria indicates very different strength of classification of coaches into the group of “experts” or “non-experts,” as, for example, the criterion “education” classifies as many as 70.4% [138] of sample (Figure 3), the criterion “experience” 48.5% [95] of the sample (Figure 2) and the criterion “success” classifies only 17.4% [34] of coaches (Figure 1) from our sample as “experts”. An overview of the separate classifications according to each expertise criterion shows that the most distinguishing criterion is “success,” as it classifies the smallest group of coaches of our sample as “experts,” thus proving to be the most demanding criterion for determining expertise. This is understandable and expected, as achieving high competitive results of their athletes requires a high level of both implicit (experience) and explicit (education) knowledge from the coach, which usually, but not necessarily, is realized in high competitive results.

However, from the analysis of our sample of gymnastics coaches, we can also see that “success” is not always the ultimate measure of expertise, as the distribution and ranking of coaches based on the GEF equation (Table 3) shows that only 13.8% [27] of coaches in our sample meet all three criteria simultaneously and can therefore be defined as “expert” coaches. As a result, seven [7] coaches who reached the “international” coach level (Figure 1) were classified as “Proficient” coaches (2nd highest level of expertise), with detailed analysis showing that coaches with insufficient education [3] had an average of more than 21 years of domain-specific experience and those with insufficient experience [4] had a high level of education. Although that this suggests that a compensatory effect between two forms of knowledge (explicit and implicit) is possible to achieve high competitive results (success), is usually, as shown by the results of our sample, achieving a high level of “success” also requires a defined level of achievement of both remaining criteria (experience, education).

Although, as its visible from results of GEF_sum_ (Table 3), the amount of domain-specific experience and high level of education of coaches alone is also not sufficient to achieve high competitive results of their athletes. Namely, as many as 86.5% of coaches [45] classified as “Proficient” coaches who did not reach the international level coach (“success” criteria) have an average of more than 21 years of domain-specific experience and an average level of education is the same as for coaches classified as “Expert”. This could of course be explained by the high level of competition in gymnastic disciplines and the global spread of gymnastics, or by the poor material working conditions (e.g., poor gym halls, insufficient equipment, etc.) in which coaches work. However, since they conduct training in comparable material conditions and compete in the same global competitive environment as coaches classified as more successful coaches (Figure 1) it is more appropriate to understand this phenomenon in accordance with Ericsson (2018b) claim that “*extensive experience* (and high education) *in a certain field does not always lead to a professional level of achievement* (results)*, as it is not always associated with a deliberate improvement in performance*”. For this reason, the developed GFE model predicts that a coach must achieve all three criteria of expertise simultaneously to be recognized as an “Expert” coach in gymnastics.

### 6.2 GEF in light of the Collins-Evans three-dimensional model of expertise

**Figure 5:**
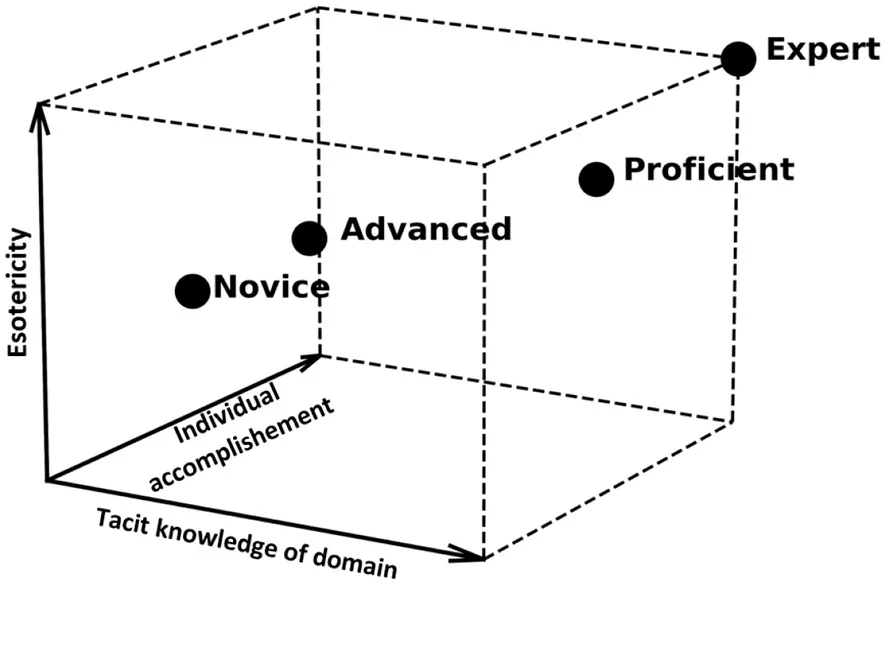
Positions of expert level groups of coaches calculated using the GEF equation in three-dimensional space of expertise developed by Collins and Evans (2018). Source: adapted from Collins and Evans (2018).

The developed GEF therefore follows the fundamental theoretical emphasis of Ericsson (2018a) that an expert must have long-term and intensive experience with practice and education in a specific field and also upgrades it with the additional element of Collins and Evans (2018)'s expertise meta-criteria's which, in addition to experience and knowledge specific to the field, also define the criteria of achieved “results” (success). Moreover, if the groups of coaches developed with the GEF model were placed in the Collins and Evans's (2018) three-dimensional model of expertise (Figure 5), which authors developed based on a sociological-philosophical perspective, the group of “Expert” coaches are placed at the extreme edge of the three-dimensional space and therefore be defined as those who (1) have high domain-specific expert explicit knowledge (education) with proven practical reproduction (achieved results) (“z” axis), (2) acquired in the so-called “esoteric” or narrow field of gymnastics training expertise (closed experience) (“y” axis), (3) from the field of published sources (written, video...), through linguistic interaction (with a group of other experts) and full participation in the field of theory and practice (creation also a new knowledge) of domain-specific expertise (experience) (“x” axis). The other defined groups of coaches (“Novice,” “Advanced,” “Proficient”) are located somewhere within the three-dimensional space, with a tendency to approach the extreme edge (closer to the group of “Expert” coaches), whereby the three-dimensional spatial distance to the group of “Expert” coaches would decrease depending on the increasing value of GEF_sum_. However, this approximation to the extreme edge is not uniform because the differences in the values of individual criteria/dimensions between groups change unevenly. Accordingly, we can observe in Figure 4 that the level of experience increases relatively evenly, while the level of education (explicit knowledge) increases relatively evenly from the “Novice” group to the “Proficient” group, and the difference between “Proficient” and “Expert” groups is very small, and that the practical reproduction of expertise measured by accomplished competitive results (success) is the one that makes largest distance of three groups from the “Expert” group in three-dimensional space. The position of an individual group of coaches in the three-dimensional space will also be strongly influenced by the dimension of “esotericity,” i.e., openness/closedness of experience, as coaches from higher quality groups have increasingly better and greater access to closed environments for the exchange of expert experience and knowledge (e.g., international training camps for coaches, highest level international competitions...), with the assumption that the greatest jump toward the extreme edge of this dimension is between the “Advanced” and “Proficient” groups. This assumption can be supported by the findings of the authors, who claim that coaches at higher competitive levels have more opportunities to learn than coaches at lower competitive levels, which contributes to the improvement of their expertise and competences (Rodrigues et al., 2009; Mesquita et al., 2011).

### 6.3 GEF in relation to the DM behaviors of coaches

Coaches' expertise, as defined and classified by GEF, provides a rational basis for their decisions which are reflected in the technical, tactical, psychological, physical and all other aspects of athletes' preparation and as such form the basis for the development of the athlete's career success. Harvey et al. (2015) claim that coaches' DM represent defining element of coaching expertise, while other authors argue that coaching is, fundamentally a DM process (Abraham and Collins, 2011; Lyle and Muir, 2020). Consequently, DM can be defined as the fundamental method by which coaches implement all aspects of the training process (Abraham et al., 2006; Kolar and Tušak, 2022; Wilson and Kiely, 2023). When examining the correlations between the measured expertise criteria (experience, education and success) and the structure of DM styles (Table 2), we found that only the rational DM style (DMSR) is statistically significantly positively correlated with all three success criteria, while of the other measured DM styles, only the spontaneous DM style (DMSS) is statistically significantly, but negatively correlated with the level of explicit knowledge (education) of the coaches in our sample. The assumption that more experienced, more educated and more successful coaches are also more rational in making decisions was therefore also verified by analyzing the correlations between the obtained GEF_sum_ values and the measured DM styles. The results of the correlation analysis presented in Table 5 confirm this assumption, as the established relationships show that a higher value of GEF_sum_, which indicates a higher level of expertise, is statistically significantly positively correlated (*p* = 0.000) with a greater use of the rational DM style (DMSR).

**Table 5:** Correlations between GEF and DM styles of coaches.

Parameter	DMSR	DMSI	DMSD	DMSA	DMSS
GEF_sum_	**0.249** ^**^	0.015	−0.045	−0.071	−0.014

^**^Correlation is significant at the 0.01 level. Statistically significant correlations are indicated in bold.

Additionally, we also examined which DM style (predictor variables) best explains the variance of GEF_sum_ (criterion variable) of the selected sample of gymnastics coaches. The results of the regression analysis (stepwise method) showed a low (5.7%) but statistically significant (*p* = 0.000) explanation of the GEF variance with the variable rational DM style. Other DM styles do not explain statistically significant variance of GEF_sum_ variable.

**Figure 6:**
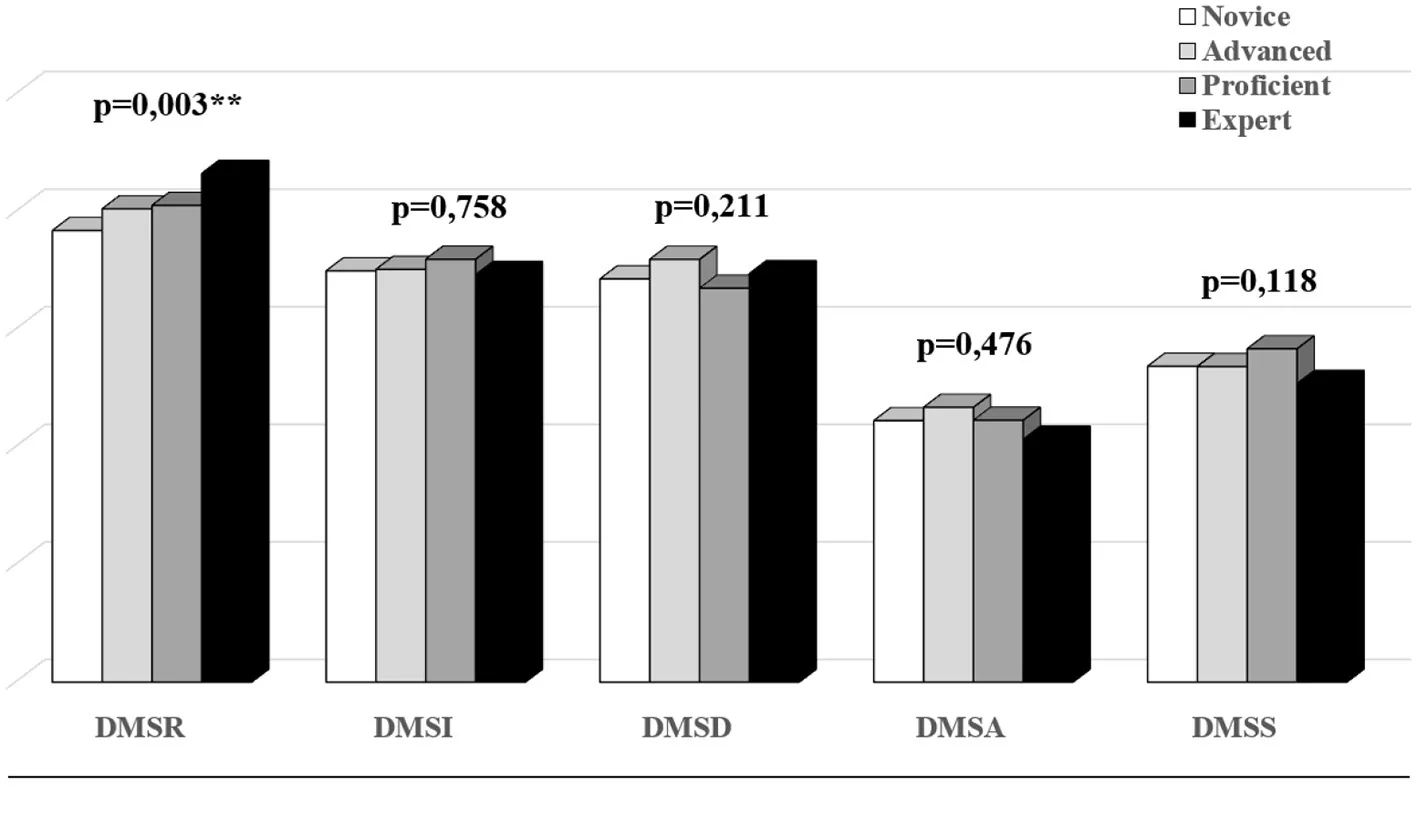
Analysis of differences in DM styles between different expert level groups of coaches calculated on GEF equation. **Correlation is significant at the 0.01 level.

Moreover, the analysis of differences in the use of individual DM styles between groups of coaches classified by level of expertise based on GEFsum showed that the groups differ statistically significantly (*p* = 0.003) only in the use of the rational DM style (Figure 6). Furthermore, the analysis of differences between individual groups of coaches in using the rational DM style shows that the “Expert” group is statistically significantly different from the “Novice” (*p* = 0.000) and “Advanced” (*p* = 0.007) groups at a 1% risk level and from the “Proficient” group (*p* = 0.022) at a 5% risk level, while the difference between the “Novice” and “Proficient” groups (*p* = 0.050) is at the border of the 5% statistical risk level. The results of all analyses on the use of individual DM styles by coaches at different levels of expertise (Figure 5 and Table 5) or in relations to the individual criteria's of expertise (Figures 1–3 and Table 1) therefore show that the higher an individual coach is ranked according to GEFsum, or the more he/she meets individual criteria of expertise, the more rational he/she is, or the more he/she uses a rational DM style when making decisions. Based on the above, we can therefore conclude that rationality is one of the defining elements of expert coaches.

Additionally, we can draw at least three more conclusions from observing the results of analyses of the use of coaching DM styles among different groups of coaches (Figure 6). First (1), from an overview of the average structure of DM styles of individual groups of coaches, we can see that coaches from the “Advanced” group use more rational DM style than the “Novice” group, with the difference being statistically significant at a 10% risk level (*p* = 0.067), and they also use more dependent and avoidant DM style (the differences are not statistically significant), which can be understood as them being more rationally aware of their lack of knowledge and experience and therefore being more dependent on the opinions of their professional environment in making decisions and thus avoiding making decisions individually more. Second (2), coaches from the “Proficient” group, who are characterized by a high average amount of experience (more than 20 years; Table 3), are characterized by being the most intuitive (DMSI) and spontaneous (DMSS) and least dependent (DMSD) decision makers, which indicates the ability to make quick decisions in time-constrained situations where there is not much time for negotiations and agreements, which is likely encouraged by their extensive experience (Klein et al., 2010). Finally (3), the most rational (DMSR) coaches classified in the “Expert” group are also the least avoidant (DMSA) decision makers, which indicates a high level of knowledge and experience and a high level of confidence in their expertise (Giske et al., 2013; Noh et al., 2018; Kolar et al., 2025b). Also, the coaches in the “Expert” group are characterized by a relatively high level of use of the dependent DM style (DMSD), which indicates that they are able to check and coordinate their judgments within a wider professional team (Kolar et al., 2025a).

### 6.4 GEF in light of the Dunning-Kruger effect curve

**Figure 7:**
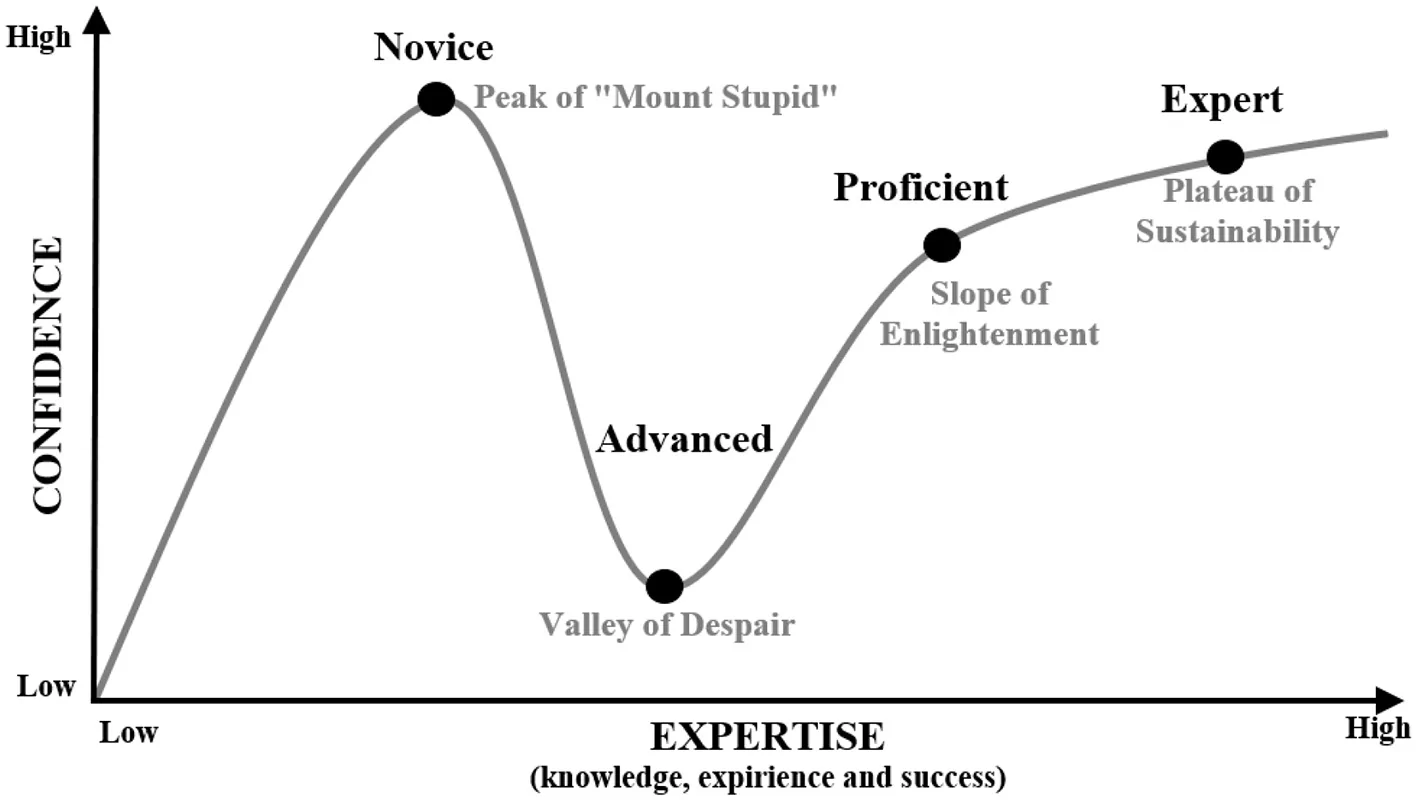
Positions of expert level groups of coaches calculated using the GEF equation on Dunning–Kruger effect curve. The ratios on the “x” axis (EXPERTISE) are calculated based on normalized average values (values for the “Experts” group have a value of 100% for each criterion) obtained when forming individual groups with the GEF equation, which, after normalization, are summed into the total relative value of expertise and therefore reflect the actual distances (on the “x” axis) in expertise between individual groups of coaches.

All insights obtained from the analysis of differences in the use of DM styles among different groups of coaches (Figure 6) can be explained relatively clearly using the theory of the “Dunning-Kruger Effect” (Kruger and Dunning, 1999). The Dunning-Kruger effect (hereinafter DKE) describes a behavioral pattern in which people who lack knowledge and/or experience in domain of judgment and DM are likely to overestimate their abilities, in part also because they lack the necessary skills to recognize errors. DKE expose discrepancy between competences and self-confidence caused by limited self-awareness (Whitfield, 2025), which can be display on a DKE curve. Figure 7 shows the DKE curve on which the groups of coaches defined by the GFE equation are placed. The distribution of individual groups of coaches on the DKE curve was largely conditioned by the calculated average value of experience, formal knowledge (education) and success of each group (Table 3), while the interpretation of the expected behavior of members of an individual group is possible to describe primarily by observing the average structure of DM styles of an individual group (Figure 6).

In Figure 7 we see that the coaches in the “Novice” group are at the so-called peak of “Mount Stupid”. As we can see from Table 3, they have on average only 4.8 years of experience and on average have not reached the level of high education. In our case, the “Mount of Stupid” is a concept that describes the phenomenon that coaches with little knowledge or ability tend to overestimate themselves and represents a specific phase of the “Dunning-Kruger effect,” where the word “stupid” itself is understood in accordance with the definition that Stanovich (2009) use in his book as the “*tendency to make bad decisions or mistakes due to negligence*”. This group of coaches are characterized by having enough experience to understand the basics of sports training in gymnastics, but they certainly have not yet developed the ability to understand the comprehensivity and complexity of the training process, so they are unable to recognize the possibility that their judgements and decisions can be wrong. They are biased due to excessive self-confidence in judgments and decisions, which can be seen (Figure 6) in the low use of rational (DMSR), relatively high values of the use of intuitive (DMSI) and spontaneous (DMSS) DM styles, and, for their level of competence, relatively low values of use of dependent DM style (DMSD). This means that they make snap decisions based on System 1 judgments (intuition) that operate automatically without conscious thought and occur quickly when coaches notice a situation, which needs to be solved. They are consciously aware of the solutions, but not of the process and use of information (tacit knowledge/experiences) to generate them (Simon, 1992). Thompson et al. (2011) hypothesize that the initial intuitive perception is accompanied by a metacognitive experience, which they call the sense of correctness of the decision (e.g., good feeling, feeling of rightens). Metacognition is the minds way of checking his own work (Whitfield, 2025) and represents a second-order computation or self-assessment by which people judge their own self-confidence and judgement (Jansen et al., 2021). Mosier et al. (2018) explain that experts not only monitoring “what” but also “how” they are thinking and whether it is appropriate for the situation in hand, and for this is responsible metacognition. In previous study's authors confirm that there are a miscalibrations in ability to judge own performance across a large variety of domains, mostly because of lack of experiences and knowledge (Dunning et al., 2004; Ehrlinger et al., 2008). Frith (2012) wrote that metacognition refers to the processes by which we monitor and control our own cognitive processes, including DM. Dunlosky and Metcalfe (2009) define metacognitive monitoring as the assessment or evaluation of the process or current state of a particular cognitive activity, and metacognitive control as the regulation of a cognitive process that is being carried out. The result of monitoring a process in progress can be (1) stopping the process (error), (2) deciding to continue (good feeling), or (3) changing or correcting its implementation (ambiguity). Self-monitoring (self-assessment), enabled by the metacognitive system, allows an individual to evaluate intuitive cognitive thoughts and check whether the chosen solution is appropriate for a particular situation, as well as to check for possible errors in previous thinking (Andryauskas, 2016). So, why “Novice” coaches are not capable to make a good self-assessment and why metacognition is not helping them when they judge and making decisions? Shea et al. (2014) argue that metacognition is characterized by operating automatically at an unconscious level and is characterized by the control of cognitive processes in individual DM. This process is said to be based on the same information as the DM process, which is said to operate at a lower cognitive level and to control it and, if necessary, identify discrepancies with the desired consequences (errors). Similarly, Fleming and Daw (2017) note that metacognition is best explained by a second-order computational model in which metacognitive processing is related to, but separate from, first-order processes (cognitive decision styles) that underlie cognitive functioning. Therefore, if the coach has the same information available for cognition (the DM process) and metacognition (the control of judgment), his metacognitive sensitivity to distinguish correct from incorrect responses should be equal to his cognitive sensitivity to the task. Or as McIntosh et al. (2022) explained, “*if a person does not have enough information to know the correct answers* (cognitive DM process)*, he will not have enough information to know which of his answers are correct* (metacognitive control)”. Since the “Novice” coaches lack sufficient knowledge and experience, metacognitive perceptions do not recognize errors or ambiguity in judgment, so the metacognitive system signals a “good feeling,” suppresses the engagement of System 2 (rational-analytic cognitive style) and thereby confirms the initial judgment of System 1 (intuitive-experiential cognitive style), which leads to a decision that manifests itself in the form of an intuitive (DMSI) or spontaneous (DMSS) DM style. The feeling of rightness of the decision represents the level of self-confidence about the correctness of the initial (prime) decision and serves as a confirmation of the correctness of the initial (System 1 or intuitive) solution. All of the different kinds of System 1 processing, also associative and implicit learning processes, can produce responses that are irrational in a particular context if not overridden. System 2 processing thus involves inhibitory mechanisms which is one of the most critical functions of System 2 processing and override System 1 processing, which is sometimes necessary because System 1 processing is “quick and dirty” (Stanovich, 2009). Qiu et al. (2018) argue that the process of examining the outcome of an initial decision (System 1) and giving instructions that the decision needs to be adjusted (System 2 activation) is called metacognition and is usually triggered automatically. These metacognitive assessments of decision quality are important for the guidance of behavior, particularly when external feedback is absent or sporadic (Fleming and Daw, 2017). Accordingly, “Novice” group coaches, due to their lack of experience and knowledge, have so-called *metacognitive deficits* (Kruger and Dunning, 1999) and are prone to making quick decisions, unaware of their *unconscious incompetence* (Swart, 2022), which can often lead to undesirable consequences, so their decisions in the training process must be subject to continuous mentoring and supervision by coaches with more proven expertise.

A group of “Advanced” coaches are stationed on the DKE curve in a place called “Valley of Despair” (Figure 7). Table 3 shows, that coaches from this expertise group have on average 9.55 years of experience and on average have completed the first level of tertiary education (1st Bologna level). The increase in the scope of implicit (experience) and explicit (education) knowledge of coaches from the “Advanced” group compared to coaches classified in the “Novice” group (observed on the “x” axis of the curve in Figure 7) causes a drastic decline in the self-confidence (observed on the “y” axis in Figure 7). The “Valley of Despair” represents the lowest level of self-confidence and highest level of uncertainty in the work of coaches, which is a consequence of increased rationality (DMSR) and thus awareness of the full complexity of the training process, acquired with progress in experience and knowledge. Doubts about the sufficiency of experience and knowledge to manage this complexity are reflected in the increase in decision avoidance (DMSA) and the emphasized dependence (DMSD) on opinions and guidelines that coaches from the “Advanced” group seek in the professional environment. The above is clearly evident from the changes in the structure of DM styles shown in Figure 6. Coaches classified in the “Advanced” group become able to recognize gaps in their expertise and realize that there are things they do not yet know or understand. They feel overwhelmed and insecure, which fosters the so-called “imposter syndrome” (Clance and Imes, 1978), which is a natural and healthy feeling that can push the boundaries of their competence, as it offers a sense of humility and motivation to learn (Swart, 2022). In this way, they enter a phase of so-called conscious incompetence (Swart, 2022), which is a fruitful starting point for further learning and practice. Positive results and feedback allow them to increasingly recognize their competence in the acquired skills and slowly slide into the conscious competence zone (Swart, 2022), which is enabled primarily by positive feedback obtained from higher-level experts or/and from positive outcomes of their work with athletes. Due to the progress in knowledge and experience, their metacognition also develops, which becomes their main “enemy” in DM processes, as conscious awareness constantly reminds them of the possible inadequacy of their judgements and decisions given the perceived complexity of the sports training process. Therefore, this group of coaches, although they can independently manage the training process, will still need a lot of mentoring, which, given the structure of their DM styles, they will now seek out on their own from more professionally skilled coaches.

Coaches from a group “Proficient” are placed on a DKE curve on a place called “Slope of Enlightenment”. Coaches from this expertise group have in average more than 20 years of experience and in average completed 1st Bologna level of high education (Table 3). They are characterized primarily by a high level of implicit knowledge, encouraged by many years of experience in gymnastics training and participation in international competitions, upgraded with explicit knowledge acquired in the school process, and probably also through informal theoretical education and independent learning. A large and relevant “knowledge base” enables them to validly and effectively use intuitive reasoning (System 1), which is also evident from the emphasized manifestation of an intuitive (DMSI) and spontaneous (DMSS) DM styles (on average the highest among all groups of coaches; Figure 6). These coaches have fully developed conscious competence, and based on the structure of DM styles, it is also possible to conclude the same about the development of *unconscious competence* (Swart, 2022), where coaches no longer think about their knowledge and its application, but make decisions automatically and quickly when necessary. Their self-confidence is justifiably high, and they also trust their metacognition, which is now developed to such a level that it communicates to them relevant feelings related to initial/intuitive decisions. Their DM style structure (Figure 6) shows that compared to the previous group of trainers (Advanced), they did not progress in rationality, the progress of which is evident mainly in the transition from “Novice” to “Advanced,” but mainly in manifest forms of DM, which indicate the operation of System 1 and the so-called *expert intuition* and a simultaneous reduction in dependence and avoidance in DM. According to Nash et al. (2023), experienced coaches tend to develop more efficient cognitive routines, recognize patterns in complex situations, and make decisions with greater confidence, which is supported by reflective experiences accumulated throughout their careers (Trudel et al., 2016). The aforementioned changes comprehensively indicate a significant increase in expert knowledge and, above all, self-confidence in their decisions.

The “Expert” group of coaches from our sample is positioned on the DKE curve at a point called the “Plateau of Sustainability” and has developed and achieved complex coaching mastery. Coaches in the “Experts” group have an average of more than 26 years of experience and an average of more than 4 years of completed higher education (Table 3). These coaches also demonstrate a distinctly high value on the international performance of the athletes they coach, which indicates the high reproductive value of their knowledge and experience. They mostly hold the position of national team coaches and thus assume responsibility, duty and authority for the strategic national development of gymnastics. They also act as teachers and mentors to the coaches from the “Novice” and “Advanced” groups. This group of coaches is, on average, by far the most rational and least evasive in DM processes, while also demonstrating an appropriate level of cooperativeness. They are also wary of making quick decisions, although they do not prefer them, and probably only make them when circumstances require them. Their metacognition is “well-educated” and trustworthy, so these coaches are confident and prudent in their DM. They are masters of expertise in gymnastics coaching with proven results from their work.

### 6.5 GEF in light of the conceptual framework of coaches' DM in conventional sports

The developed conceptual framework foresees three types of decisions; (1) strategic, (2) tactic and (3) operational, which are interconnected within the training process (Kolar et al., 2025a). As authors wrote the strategic type decisions are at the most general level directs the training process over a longer period, and its implementation is determined by the tactical type decisions, while the operational type decisions solve sudden emergency situations and problems that arise when implementing the tactical type decisions in timeframe of everyday training or competition (Kolar et al., 2025a). According to the developed framework, the appropriate adoption of all three types of decisions requires an extensive amount of knowledge and experience, with explicit knowledge being favored in the formation of strategic decisions, implicit knowledge in the adoption of operational decisions, while a comprehensive “knowledge base” consisting of both acquired theoretical knowledge and practical experience is recommended for the formation of relevant tactical decisions. Consequently, coaches should use a rational and dependent DM style (System 2) for strategic decisions, an intuitive and spontaneous DM style for operational decisions (System 1), and an appropriate combination of the aforementioned styles for tactical decisions, with the rational and intuitive DM style prevailing.

Therefore, if we place the insights from the developed GEF model in the context of the conceptual framework of coaches' DM, we can see that only coaches from the “Expert” and “Proficient” groups are those who are considered to be sufficiently qualified (in terms of the adequacy of knowledge and experience) and have an appropriate DM behavior to independently and professionally relevantly make all three types of decisions. For the efficient and successful adoption of all three types of decisions, it is also important that, due to the acquired extensive “knowledge base,” they also have a developed and capable metacognitive sensitivity that is capable of recognizing errors in cognitive activity (in the DM process), because as Fleming and Daw (2017) predict, there is a close connection between metacognitive assessments of confidence and strategic control of DM. Given the structure and requirements for making individual types of decisions, according to the resulting groups of coaches with GEF, coaches from the “Expert” group are more suitable for making strategic type decision and coaches from the “Proficient” group for adopting operational type decision, while for forming tactical type decisions, a group with a balanced structure of both mentioned quality groups of coaches is most suitable. Coaches from the “Novice” and “Advanced” groups, given the described characteristics, are not suitable for independently making any of the above-mentioned types of decisions in a process of training and competitions.

## 7 Conclusions

In the present article, the authors developed a mathematical model of the General Expertise Factor, for the development of which, experimentally collected data from a sample of gymnastics coaches were used. The development of GEF is based on the premise that expertise in a particular field is multidimensional, whereby dimensions or criteria of domain-specific explicit knowledge, experience, and confirmed (sports) results must be simultaneously achieved for an individual coach to be recognized as an expert of the highest rank. The model assumes that coaches can be divided into four levels of expertise based on the calculation of the GEF equation, with statistically significant differences between individual groups being found for all three selected expertise criteria. It was also found that all three criteria together explain the majority of the GEF variance. Moreover, the comparation of the resulting quality groups of coaches according to their average DM behavior reviled that the groups differ significantly in the use of a rational DM style, with rationality in DM consistently increasing in line with the increasing competence or expertise of the representatives of each quality group. We also checked the compliance of the developed GEF model with other, already established models in the areas of expertise and DM and found a high level of consistency.

Based on the findings of our research, we can conclude that the developed GEF well stratifies gymnastics coaches into different levels of competence and expertise and that rationality is one of the important factors that, together with knowledge, experience and achieved success, represents a defining element of coaching expertise. It should also be emphasized that in our case, rationality is understood as “bounded” due to the internal and external constraints of coaches as decision-makers, “procedural” from the perspective of considering the logical and sequential DM process and “extended” from the perspective of using external (knowledge of other experts) resources in the process. In line with our findings and the fact that the management of the training process is inextricably linked to DM, we conclude with the thought of Stanovich (2009), who claimed that good decisions do not necessarily depend on intelligence, but primarily on rationality.

### 7.1 Practical implications

The practical value of the presented GEF lies primarily in its potential use in determining the criteria for the suitability of individual coaches for various responsible roles in national coaching hierarchies and in identifying the level of competence of individual coaches for independent management of the training process. Regardless of the fact that the GEF model was developed exclusively on the basis of data obtained from coaches of various gymnastic disciplines, the underlying logic of the model is general enough that it can also be used in other sports by adjusting the calculated norms for each criterion, depending on the complexity of the individual sport.

The findings of this study also offer practical guidelines for (1) designing progressive pathways for coach education, (2) structuring mentoring and supervision of coaches, (3) assigning coaching responsibilities, (4) forming coaching teams, and (5) identifying areas in which individual coaches need additional education, practical experience, or exposure to higher-level competition.

### 7.2 Limitations and possible future research directions

This study has the following limitations that should be considered:

- The cross-sectional design does not permit causal conclusions or the examination of changes in expertise over time;
- Decision-making styles and professional characteristics were based primarily on self-report and may be affected by recall error, social desirability, and common-method bias;
- The sample included only gymnastics coaches from Serbia and Slovenia, with an unequal national distribution and a pre-dominance of female participants, limiting generalisability to other sports, countries, and coaching systems;
- The low reliability of the spontaneous decision-making subscale limits conclusions involving this variable.
- The proposed classification and the GEF is an initial operational classification model whose external validity should be validated.

Possible directions for future research could be based on:

- Replication using independent samples of gymnastics coaches;
- Testing in other countries and coaching systems;
- Application in different individual and team sports;
- Comparison of the present dichotomous scoring system with continuous scoring;
- Examination of alternative thresholds and weighting procedures;
- Longitudinal examination of coaches' movement between GEF groups;
- Validation against independent expert ratings and subsequent athlete-development outcomes;
- Examining differences between groups of coaches formed with GEF in terms of their leadership styles, personality traits, motivational structure, and other psychometric characteristics.

## Data Availability

The raw data supporting the conclusions of this article will be made available by the authors, without undue reservation.
